# Nanoparticles: A New Approach to Upgrade Cancer Diagnosis and Treatment

**DOI:** 10.1186/s11671-021-03489-z

**Published:** 2021-05-20

**Authors:** Zhongyang Yu, Lei Gao, Kehan Chen, Wenqiang Zhang, Qihang Zhang, Quanwang Li, Kaiwen Hu

**Affiliations:** 1 Oncology Department, Dongfang Hospital, Beijing University of Chinese Medicine, Fangguyuan Rd, Fengtai District, Beijing, 100078 China; 2 College of Engineering, China Agricultural University, Tsinghua East Rd, Haidian District, Beijing, 100083 China; 3 Department of Management, Fredericton Campus, University of New Brunswick, 3 Bailey Drive, Fredericton, NB E3B 5A3 Canada; 4 Beijing University of Chinese Medicine, 11 North Third Ring East Road, Chaoyang District, Beijing, 100029 China

**Keywords:** Nanoparticle, Nano-cryosurgery, Targeted delivery, Photothermal therapy, Cancer

## Abstract

Traditional cancer therapeutics have been criticized due to various adverse effects and insufficient damage to targeted tumors. The breakthrough of nanoparticles provides a novel approach for upgrading traditional treatments and diagnosis. Actually, nanoparticles can not only solve the shortcomings of traditional cancer diagnosis and treatment, but also create brand-new perspectives and cutting-edge devices for tumor diagnosis and treatment. However, most of the research about nanoparticles stays in vivo and in vitro stage, and only few clinical researches about nanoparticles have been reported. In this review, we first summarize the current applications of nanoparticles in cancer diagnosis and treatment. After that, we propose the challenges that hinder the clinical applications of NPs and provide feasible solutions in combination with the updated literature in the last two years. At the end, we will provide our opinions on the future developments of NPs in tumor diagnosis and treatment.

## Introduction

The incidence and mortality of tumors remain high worldwide. Every year, there are nearly 14 million new cancer patients and 8 million people die of cancer-related diseases [1]. In recent years, traditional tumor treatments, such as chemotherapy, targeted therapy, radiotherapy, surgery, etc., are constantly criticized for being bogged down in progress and for many adverse reactions and unsatisfied treatment outcomes. Because of the shortcomings of traditional tumor therapies, more and more researches have begun to seek new tumor medical methods with targeting ability, effective tumor stem cell killing ability and minor adverse reactions. New tumor treatment methods include, but are not limited to, immunotherapy, targeted therapy, physical ablation, gene therapy, photodynamics therapy (PDT) and photothermal therapy (PTT) which have shown superior efficacy compared to traditional tumor therapy. The treatment methods herein all have a common feature that requires carrier cooperation. Although viruses can be used as carriers, viral vectors have been confirmed to cause insertional mutagenesis and immunogenicity [2]. Therefore, finding a safer and more effective carrier has become a top priority.

Due to nanoparticles’ small size, biosafety, drug loading, and physical properties can assist physical therapy, nanoparticles have been increasingly utilized as carriers in new tumor treatment methods. These nanoparticles-mediated therapies have virtues of multi-function, less adverse reactions and better curative effect [3]. In addition, many medical imaging technologies mediated by nanoparticles also have better clarity and accuracy, which helps accurate tumor diagnosis [4]. With the development of nanotechnology and medical technology, metals and biological materials such as gold, silver, iron, liposomes, etc. have been widely applied in the production of medical nanoparticles (NPs) [5]. At present, many researchers utilize those materials based on their physical, chemical, and/or biological properties to embed drugs, imaging agents and even genes in nanoparticles, expanding the existing field of tumor diagnosis and treatment such as drug targeted delivery, enhanced imaging, cryosurgery, PTT and PDT [6].

In addition, there is a phenomenon that most of the nanoparticles only stay in vivo and in vitro stage. However, there is a lack of literature to summarize the reasons that deter the clinical application of NPs. Therefore, this article aims to not only summarize the application status of nanoparticles in the field of tumor diagnosis and treatment, but also to find the factors that inhibit the entry of nanoparticles into clinical applications and propose feasible solutions.

## Preparation and Characterization of Medical Functional Nanoparticles

Nanoparticles commonly used in medicine can be divided into three types: metal nanoparticles, non-metal nanoparticles and composite nanoparticles according to their constituent materials and functions, and their physical and chemical properties are affected by parameters such as size and shape. Therefore, in view of the functional requirements of nanoparticles in different application directions, it is very important to choose a suitable preparation process. All the preparation methods of nanoparticles can be classified into two methods: bottom up approaches and top-down approaches. The bottom-up approach is essentially through basic units (atoms, molecules and even smaller particles can be used as the basis for assembling the required nanostructures) stacked on each other to form nanoparticles, while the top-down approach is essentially a whole solid material begins to decompose into nanoparticles [7]. Table 1 lists some examples of preparing medical nanoparticles.

**Table 1:** Typical NPs preparation method

Synthesis approach	Material	Size (nm)	Method	Features	Ref
Bottom up approaches	TiO_2_	6–33	Sol–gel synthesis	Continuous releasing of hydroxyl radicals and superoxide ions when exposed to ultraviolet rays	[8]
	Fe_3_O_4_	10	Co-precipitation	Fe_3_O_4_ can be excited by 808 nm infrared light to realize photothermal conversion	[9]
	PEG-Fe-PDA NP	25–43	Microemulsions	MRI imaging enhancement with pH activation, high photothermal efficiency and excellent biocompatibility	[10]
	Magnetite NPs	39	Hydrothermal approach	Small size magnetic nanoparticles with biocompatibility and superparamagnetism	[11]
	Au NPs	8–300	photochemical method	Enhanced medical diagnostic imaging	[12]
Top-down approaches	Cu-Sn oxides NPs	18–40.5	electrical wire explosion	Ability to produce reactive oxygen species	[13]
	Magnetite NPs	12–20	Ball milling	Small size magnetic nanoparticles with biocompatibility	[14]

**Fig. 1:**
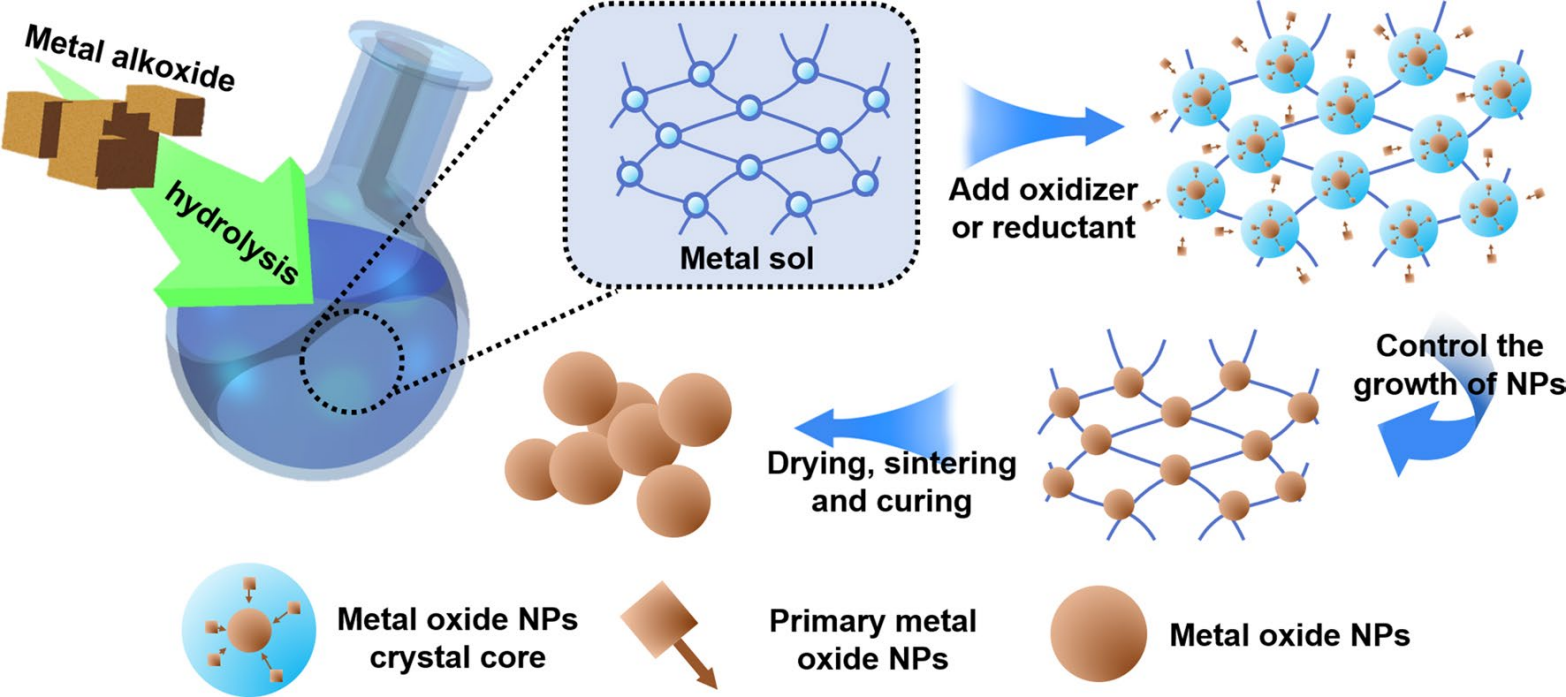
Schematic diagram of the sol–gel method

Among the three types of nanoparticles commonly used in medicine, metal nanoparticles are the most widely used. Metal nanoparticle materials include metals and metal oxides. The most commonly used preparation process for metal nanoparticles is the sol–gel (Sol–Gel) process proposed by Japanese scientist Sugimoto et al. in the 1990s, which is often used to prepare monodisperse metal oxide particles in liquid phase. The sol–gel method is a bottom-up preparation process. The main principle of this method for preparing metal nanoparticles is to form a uniformly dispersed sol of metal ions through chemical and physical means, and then form a gel through redox reaction. The metal nanoparticles generated in the gel can controllably nucleate, grow and deposit. As long as the monodispersity of the metal colloid used in the experiment, the concentration relationship of the metal ions and the oxidizing/reducing agent are controlled, the size of the synthesized metal nanoparticles can be controlled. Figure 1 is the schematic diagram of the sol–gel method.

Commonly used bottom-up methods for preparing metal nanoparticles include co-precipitation, hydrothermal approach, and photochemical method. The co-deposition method is a process of nucleation, growth and aggregation in a liquid environment at the same time. When the solution is oversaturated, a large number of small-sized particles insoluble products are obtained [15]. The hydrothermal method is a process performed in a liquid environment to control the morphology of the resulting nanoparticles by controlling the vapor pressure applied to the material in the solution. In addition, there are some top-down methods for preparing metal nanoparticles, such as electrical wire explosion and ball milling. The principle of electrical wire explosion is that in the process of electric explosion, the metal atoms are evaporated and quickly cooled in the electrolyte to form oxide nanoparticles. By controlling the electrolyte composition and current intensity, finer and uniform nanoparticles can be controlled. Ball milling is a method of quickly and large-scale production of nano-particles with controllable size using machining tools such as milling planetary gears by selecting appropriate grinding time and related equipment process parameters. In addition to metal nanoparticles, this preparation method can also be applied to other types of nanoparticles.

The second common type is non-metallic nanoparticles. Non-metallic nanoparticles commonly used in medicine include polymer nanoparticles, biomolecules derived NPs, carbon-based NPs, and silica nanoparticles [16–18]. Among them, silica nanoparticles are the most representative. The silica surface has abundant hydroxyl groups, which facilitates the binding of probes or fluorescent groups on the surface and therefore has more flexible functionality. The commonly used synthesis methods of silica nanoparticles are the sol–gel method and the Stöber method [19, 20]. The classic Stöber method is the simple and efficient preparation of silica nanoparticles through the hydrolysis and condensation of silicate under alkaline conditions.

With the development of nanotechnology, composite nanoparticles have been developed due to their superior functional compatibility. Metal nanoparticles have many characteristics that non-metal nanoparticles do not have, such as plasmon resonance effect (SPR), controllability in a magnetic field, etc., but metal particles are difficult to effectively degrade in the body, and excessive use has certain toxicity to cells [21]. Therefore, combining nanoparticles of different materials into composite nanoparticles through different preparation methods can achieve functional expansion. Wei et al. prepared gold nanorods (Au NRs), and then performed surface-initiated atom transfer radical polymerization (SI-ATRP) of N-isopropylacrylamide (NIPAAM) on Au NRs to synthesize near-infrared response Nano hybrids [22]. This composite nanoparticle that combines metal and polymer materials has both photothermal and near-infrared light corresponding drug release capabilities. The enveloping hydrogel shell makes this nanoparticle have better biocompatibility than single Au nanoparticles. Prakash synthesized composite NPs with Au as the core and SiO_2_ as the shell through the improved Stöber method. The inert shell of the core–shell nanoparticles is beneficial to reduce the toxicity of metal particles and improve the material stability and drug-carrying capacity of the original single metal NPs [23].

In addition to the traditional preparation methods of nanoparticles mentioned above, with the development of nanotechnology science, new requirements for ecological and environmental protection have been put forward, so new environmentally-friendly nanoparticle synthesis methods have emerged [24]. For the first time, Hajar et al. used Stevia rebaudiana as a biological reducing agent to successfully synthesize ZnS nanoparticles with a particle size ranging from 1 to 40 nm. The ZnS nanoparticles synthesized in this way have good biocompatibility [25]. According to the principles of green chemistry, Miri et al. used P. farcta (A plant belonging to Leguminosae) extract to quickly synthesize CeO_2_ NPs with a particle size of about 30 nm. This kind of nanoparticles has good biocompatibility [26].

## Nanoparticles for Medical Imaging

**Fig. 2:**
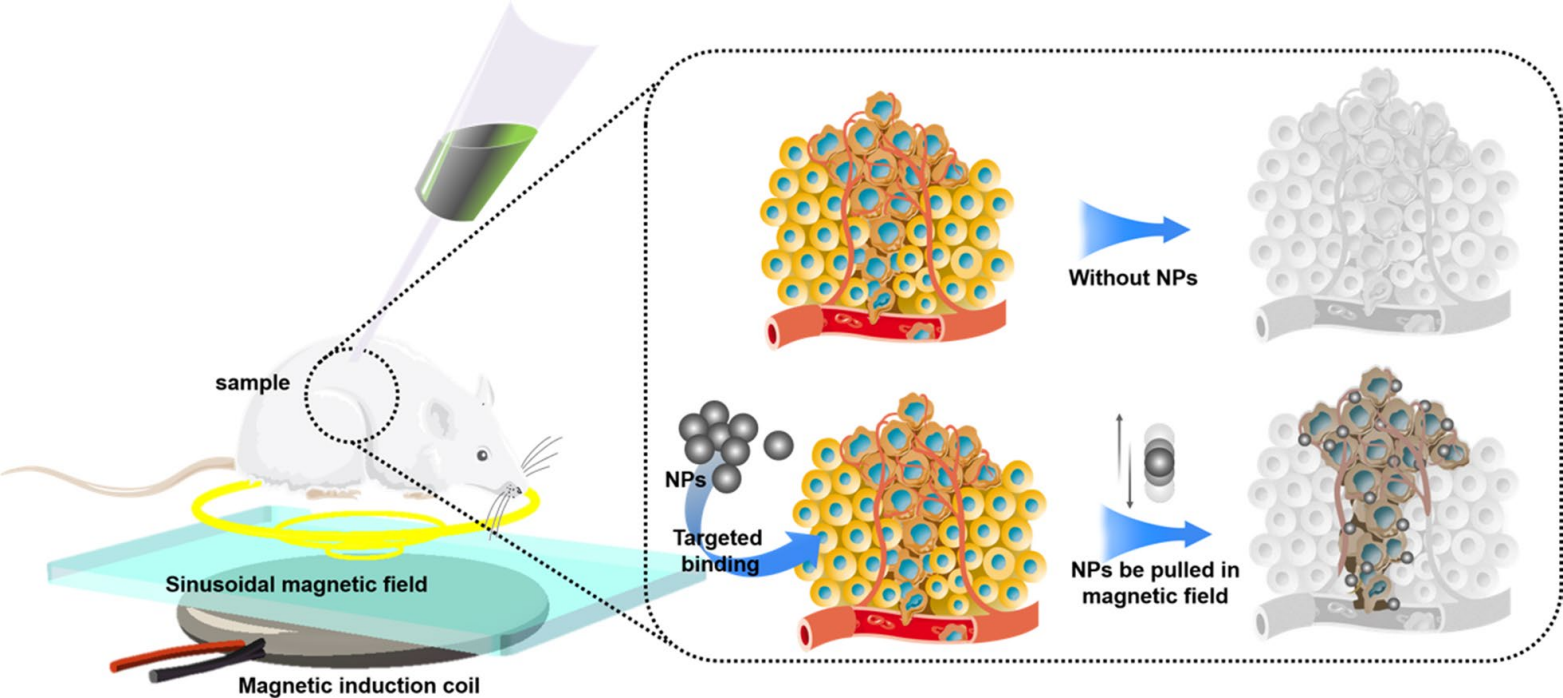
Diagrammatic illustration of imaging improved of NPs

Medical imaging plays an important role in the diagnosis and treatment of tumors. Many nanoparticles, like iron oxide NPs, have optical, magnetic, acoustic, and structural properties that can enhance imaging (Fig. 2). Some studies have shown that introducing NPs into target tissues can improve image contrast and provide better image guidance for tumor surgery and diagnosis [27]. For example, in cryosurgery, NPs can enhance the imaging quality of the tumor and ice ball edges, which helps to cover the ice balls accurately and improve the therapeutic effect [28]. In addition, most of the nanoparticles used in imaging are made of metal. According to the difference of different imaging principles, nanoparticles will also be made of different metal materials. Table 2 lists some recent examples about NPs made by different materials for medical imaging.

**Table 2:** Typical NPs platforms made by different materials for medical imaging

NPs	Size (nm)	Targeting material	Cell line	Imaging technology	Ref
MnO-TETT	6.7 ± 1.2	None	C6 glioma cells	Fluorescence/T1-MRI	[29]
PLGA-mPEG	151.1 ± 1.3	cRGD	SKOV-3 cells	US	[30]
USMO@MSNs	30–50	Dox	HeLa cells	MRI-guided chemotherapy	[31]
OINPs	300	Folate	SKOV3 ovarian cancer cells	US/PA	[32]
PEG-coated and Gd-loaded fluorescent silica	125.5 ± 9.9	YPSMA-1	LNCaP and PC3 prostate cancer cells	MRI/fluorescence imaging	[33]
SPIO/USPIO	50	None	4T1 murine breast cancer cells	MRI/MPI	[34]

*US* ultrasound, *MSNs* mesoporous silica nanoparticles, *USMO* ultrasmall manganese oxide, *GEM* Gemcitabine, *OINPs* oxygen/indocyanine green-loaded lipid nanoparticles, *PA* photoacoustic, *MPI* magnetic particle imaging, *MRI* magnetic resonance imaging, *SPIO* superparamagnetic iron oxide, *USPIO* ultra-small SPIO

Optical coherence tomography (OCT) is a non-invasive, micron-level resolution and biomedical imaging technology. OCT is useful in real-time diagnosis and surgical guidance. However, OCT cannot detect inelastic scattered light because this light is not coherent in the incident field [35]. Recently, many researches have proved the motion state of NPs can be able to change the amplitude of OCT, which may deal with this problem. Interfering with the movement of NPs through the magnetic field can cause local changes in light scattering. Some studies have pointed out that placing magnetic NPs in a magnetic field to control its motion can change the optical scattering in the area, so the originally incoherent inelastic scattered light can be detected. This new imaging method is magnetomotive optical coherence tomography (MMOCT) [36].

MRI is one of the most effective noninvasive tumor detection technology. Nevertheless, the lack of MRI signal comparison between biological background and cancer tissue often affects the clinical tumor diagnosis [37]. MRI is a scanning imaging method that measures the magnetization of hydrogen molecules in water molecules. Each anatomical structure presents a different image since the protons of each tissue cause different changes in magnetization. The visibility of images can be improved through applying more contrast agents [38, 39]. The tumor-related EPR effect widely utilized in the early detection of tumors produces great contrast enhancement ability to magnetic NPs [40]. Iron oxide magnetic NPs (IONPs) which are currently the most common MRI nanoprobe contrast agents have certain cell targeting [41]. For example, studies have found that IONPs could enter healthy liver Kupffer cells during the diagnosis of liver cancer by using MRI but will be excluded from cancer cells, resulting in low-signal healthy tissue and high-signal tumor tissue [42]. Based on recent studies, proper particle surface modification and appropriate tumor-specific bio-oligomer embedding of NPs can better fix NPs in tumors to achieve clearer imaging results and can even be used for early micro tumor imaging. For example, studies have found that AuNPs targeted for human transferrin can significantly enhance the imaging effect of brain tumors [43]. Gao et al. equipped with anti-epidermal growth factor receptor monoclonal antibody (mAb) on the basis of paramagnetic NPs probes to achieve imaging of small tumors [44].

## Nanoparticles for Targeted Drug Delivery

Although Chemotherapeutic drugs now are the most commonly used treatment for tumors, they still have the problem of poor target enrichment in malignant tumor areas and overaccumulation in healthy tissue [45]. This may cause the inhibition of cells hat divide vigorously, such as bone marrow, hair follicles, gastrointestinal cells and lymphocytes, leading to adverse reactions such as bone marrow suppression, mucositis, hair loss, and even death [46]. Targeted drug delivery which refers to active differentiation between normal cells and cancer cells for drug delivery has better efficacy and fewer adverse reactions than the conventional treatment [45].Many studies have confirmed that NPs can target chemotherapeutic drugs to tumor cells through active or passive targeting [47]. In addition, many experiments have found that NPs also play an important role in the targeted delivery of immune drugs [48].

**Fig. 3:**
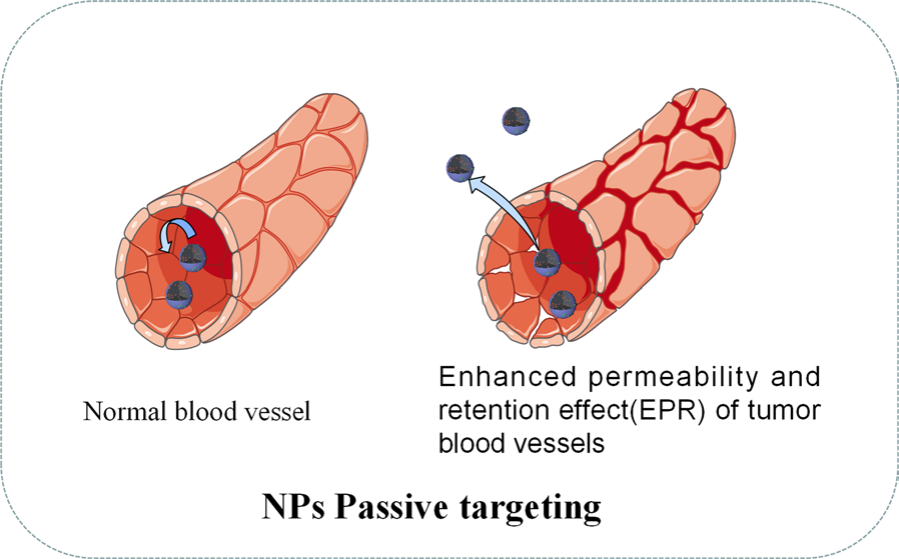
Diagrammatic illustration of passive targeting of NPs

As shown in Fig. 3, passive targeting often relies on some pathophysiological characteristics of tumor tissue, including abnormal blood vessels, temperature, pH and surface charge of tumor cells [49].For example, due to the enhanced permeability and retention effect (EPR) of blood vessels in the tumor tissue, NPs with a diameter of about 400 nm can be passively transferred to the tumor tissue [50]. However, there are many limitations on the passive targeting approach in terms of physicochemical properties of NPs such as diameter, surface charge, molecular weight, hydrophobicity, or hydrophilicity. Besides, the passive targeting technique underperforms in drug diffusion efficiency and shows insufficient EPR effect in tumor cells [51]. Due to the deficiencies of passive targeting, in recent years, most research about the drug delivery NPs has shifted to active targeting (ligand targeting). Table 3 highlights some recent examples about NPs used in drug delivery.

**Table 3:** Typical NPs platforms used in drug delivery

Agent of NPs	Vehicle	Size (nm)	Characters	Effects	Ref
DNA and RNA	Exosomes	30–100	Small size, cellular origin, flexibility to incorporate macromolecules	Carrier for DNA, RNA and micro-RNA Cross-stringent biological barriers, such as the blood–brain barrier	[52]
DOX	Polymer-lipid encapsulated manganese dioxide	170	Bioreactive and multifunctional	Downregulate TME-associated drug resistance and immunosuppression Enhancing chemotherapeutic efficacy and boosting antitumor immunity	[53]
5-FU	Au-NPs/chitosan	100–400	Natural cationic, biodegradable and biocompatible	Enhance the curative effect for hepatocellular carcinoma cells (HepG2)	[54]
Tyrosinase-related protein 2 (Trp2) peptide	Layered double hydroxide (LDH) NPs	140–150	Provoking strong cell-mediated immune responses	Adjuvant multiple tumor-associated antigen peptides	[55]
Cisplatin; ICG	PLGA	90–100	Folate targeting Controlled drug release	Promoting the apoptosis of McF-7 tumor cells NIR sensitivity	[56]
IL-2	PEGylated liposomes with anti-CD137	80	Complete absence of systemic toxicity	Inducing intratumoural immune responses Initial anti-tumor activity	[57]
FA	Magnetic mesoporous silica	213	PH-sensitive drug release	Inhibiting proliferation of HeLa cell lines higher cytotoxicity effect	[58]
PTX	Peptide H7K(R2)2-modifiediron oxide NPs	168.3 ± 2.80	few side-effects	Excellent MRI imaging Inhibiting tumor growth	[59]
siRNA	RGDfC-SeNPs	150	No toxicity Multiple tumor targeting	Carrier for siRNA Inhibiting tumor cells proliferate Promoting the generation of ROS	[60]

*DOX* doxorubicin, *5-FU* 5-fluorouracil, *FA* folic acid, *PTX* paclitaxel, *ROS* reactive oxygen species

**Fig. 4:**
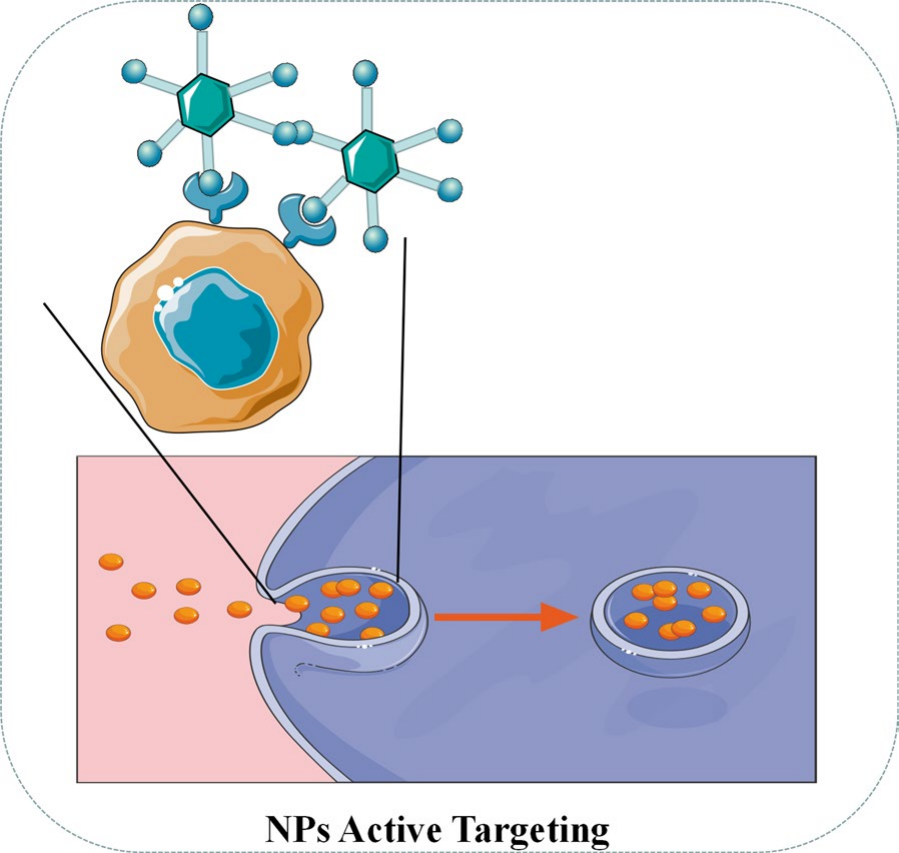
Diagrammatic illustration of active targeting of NPs

Active targeting (ligand targeting) NPs often carry some ligands of tumor-specific biomarkers [61]. As shown in Fig. 4 when the ligand contacts to the receptor on the tumor surface, NPs can be internalized by the tumor cells through receptor-mediated endocytosis, and the drugs can be released due to acidic pH and specific enzymes in the intracellular environment [62]. As for targeting ligands, folic acid, transferrin, epidermal growth factor receptor (EGFR) and glycoprotein are generally utilized in current research [62]. For example, Sandoval et al. obeserved significant drug enrichment and evident efficacy in the treatment of mice with breast cancer through EGFR-targeted stearyl NPs equipped with gemcitabine [63]. Pandey et al. found that folic acid-targeted gold NPs carrying berberine hydrochloride (BHC) can effectively deliver drugs to human cervical cancer cells expressing folate receptor [64].

In recent years, compared with chemotherapy drugs, short interfering RNA (siRNA)-mediated gene silencing therapy has been regarded as a new prospect for tumor treatment [64]. Although viruses can be used as delivery vehicles for siRNA, viral vectors have been confirmed to cause insertional mutagenesis and immunogenicity [65]. By contrast, selenium NPs are reported to have great potential as siRNA carriers, because the trace element selenium itself can reduce tumor occurrence, lower drug toxicity, and regulate immune function [66]. In addition, the surface of selenium NPs can load various tumor- targeting moieties (such as folate, hyaluronic acid and RGD peptide) to enhance tumor targeting ability [67]. Xia et al. reported that selenium NPs (RGDfC-Se@siRNA) targeted by RGDfC peptide have excellent ability to target HeLa cervical cancer [60]. Meanwhile, because RGDfC can specifically combine with α_v_β_3_ integrin which is highly expressed by a variety of tumor cells, RGDfC-Se@siRNA NPs can be reused for targeted drug delivery for a variety of tumors [68]. In terms of structure, RGDfC-SeNPs with positive charge can tightly package negatively charged siRNA through their electrostatic interaction [69]. Through animal experiments, RGDfC-Se@siRNA NPs show the ability to efficiently enter tumor cells through clathrin-associated endocytosis. In tumor cells, it can quickly release siRNA and efficiently silence related genes and promote the generation of reactive oxygen species (ROS) to inhibit tumor cells proliferate and promote tumor cells apoptosis [69]. Additionally, multiple SeNPs have demonstrated excellent biological safety and have no obvious toxic damage to liver, kidney, heart, lung, spleen and other major organs of mice [60, 70, 71].

At present, although there are many NPs used in targeted drug delivery, most applications still remain in the stage of cell or animal experiments, lacking potent clinical application support. In addition, many NPs are administered intratumorally, which limits the scope of NPs applicable to tumors and lacks special NPs drug delivery tools and other drug delivery methods.

Therefore, exploring a better way to administer NPs may be a direction for the future research of targeted drug delivery NPs. According to the existing academic journals, vascular interventional administration may be a feasible way. In the assumption, first locate the position of tumor-feeding blood vessel with the help of imaging, and then use a guide wire to introduce NPs directly into the tumor-feeding blood vessel and control the movement of the NPs in a small range by applying a magnetic field simultaneously. Therefore NPs can be fixed at the proper position without being influenced by blood flow in the vessel. Otherwise, NPs targeted for drug delivery only have certain limitations. Targeting NPs will affect the systemic distribution of chemotherapeutic drugs and reduce the effect of chemotherapy on free tumor cells and micrometastasis. If they are equipped with targeted drugs, the targeting effect tends to be enhanced whereas the improvement is not evident based on the existing studies.. In addition, anti-tumor drugs are unlikely to eliminate all tumor stem cells by themselves. Nevertheless, physical therapy based on the physical characteristics of NPs tends to be more effective against the tumor stem cells. Therefore, multifunctional NPs targeting drug carriers tend to be advisable in the future, such as cryosurgery, photothermal therapy (PTT) and photodynamics therapy (PDT) etc., to form multi-functional NPs for tumor treatment.

## Nanoparticles for Cryosurgery

**Fig. 5:**
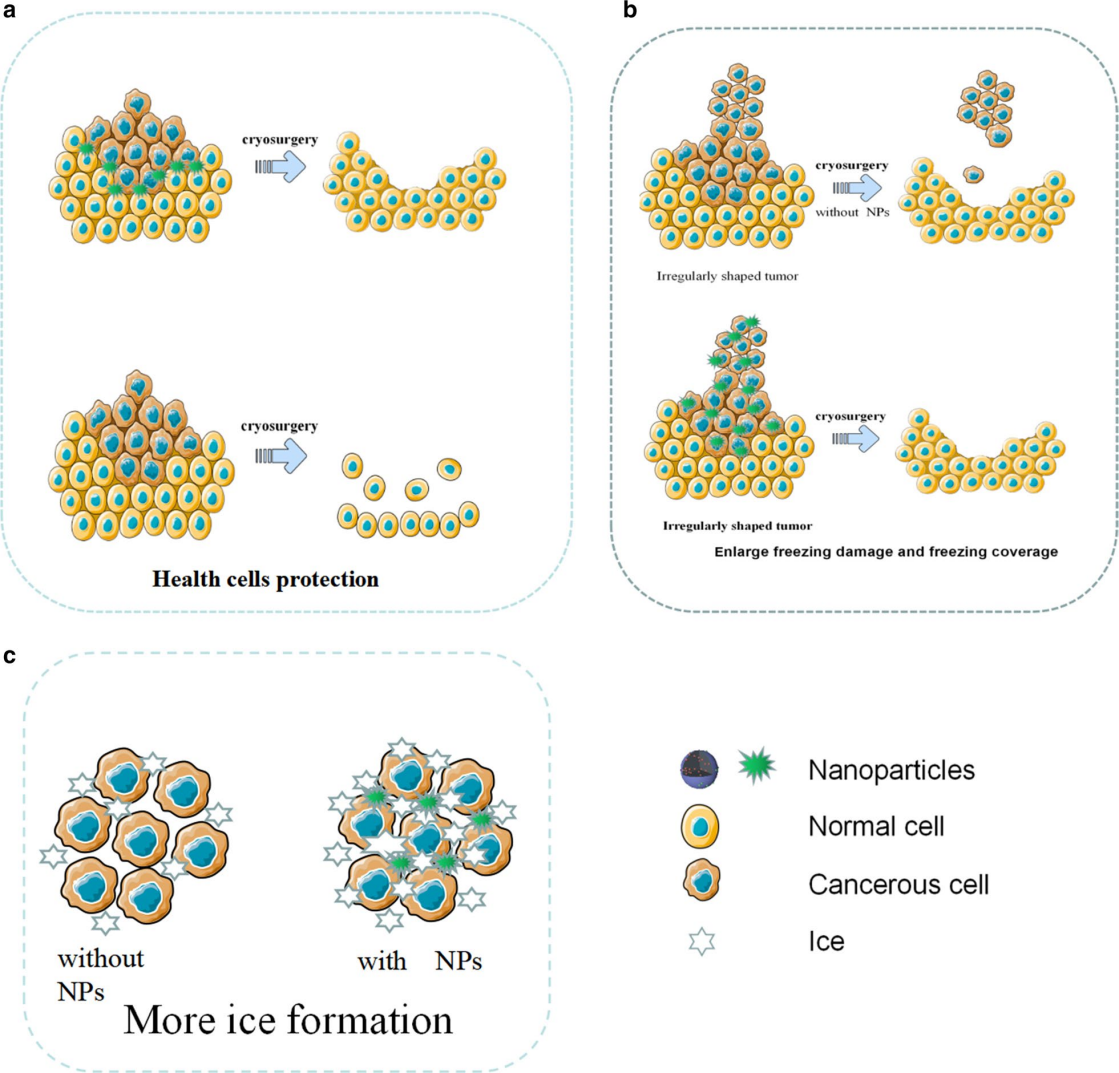
Diagrammatic illustration of NPs for cryosurgery. **a** NPs protect health cells during cryosurgery. **b** NPs enhance the freezing damage and control the freezing coverage. **c** With the help of NPs, more ice has been formed

Cryosurgery, the technique of destroying tumor tissue by freezing, has the advantages of low invasiveness, low cost, less intraoperative bleeding and less postoperative complications, but there are still disadvantages such as insufficient freezing efficiency and freezing damage to surrounding tissues [28]. Although protective agents such as antifreeze protein (AFP-1) have been utilized to assist cold ablation, the effect is still not ideal [72]. With the development of nanotechnology, the concept of nano-cryosurgery was proposed. The basic mechanism of nano-cryosurgery is to introduce NPs with specific physical or chemical properties into tumor tissues. By utilizing the properties of NPs, not only can the efficiency and effectiveness of freezing be improved, but the range adjustment and the direction of ice ball formation can be also controlled. Thus, the nano-cryosurgery is capable of killing tumor tissue and preventing surrounding healthy tissue from being frozen simultaneously [73]. The advantages of nano-cryosurgery have shown in Fig. 5.

In cryosurgery, intracellular ice formation is the key to tumor cell damage. Meanwhile, research proves that NPs can effectively induce intracellular ice formation [28]. NPs as external particles can induce heterogeneous nucleation. Studies have found that tissues enriched with NPs freeze faster than conventional tissues and are more prone to heterogeneous nucleation. Under the same freezing conditions, ice formation of tissue with NPs is easier, indicating that NPs can significantly increase the speed and probability of ice formation in cells, which can kill tumor cells more effectively [74]. In addition, NPs with metal oxide will significantly improve the thermal conductivity in tumor tissue. For example, Liu and Deng compared the temperature response curve of pork tissues with and without NPs. They found that the tissues containing NPs cooled rapidly, and the lowest temperature could reach 115 ℃, which was much lower than that of the control group without NPs.

Since tumors are usually irregular in shape, the ice crystals produced by traditional cryosurgery tend not to cover all tumor tissue. Compared with traditional cryosurgery, the nano-cryosurgery can deal with the problem easily. Because NPs can permeate into the intracellular fluid and have good physical properties like thermal conductivity, it is possible to control the growth direction and direction of the ice ball by the distribution of NPs [73].

In cryosurgery, insufficient freezing may not completely destroy tumor tissue, and excessive freezing may damage adjacent healthy tissue. Especially when the tumor is in close contact with fragile organs, its location is deep, or its shape is irregular, the damage to the healthy tissue can be particularly serious. In recent years, phase change materials (PCMs) made from NPs have demonstrated excellent protective potential for surrounding healthy tissues during cryosurgery [75]. For example, Lv et al. microencapsulated phase change NPs with large latent heat and low thermal conductivity through liposomes, and before cryosurgery, injected microencapsulated phase change NPs into healthy tissues around the tumor and found that avoided low temperature damage to healthy tissue [76].

Although NPs have been widely used in cryosurgery, there are still a series of deficiencies. First, it is still unable to control NPs in vitro, which results in uneven distribution of NPs in tumor tissue and unsatisfactory expected function. Secondly, although there are a variety of magnetic nanoparticles, the actual effect of in vitro magnetic field control NPs is still not ideal. In addition, the nano-cryosurgery is lack of clinical experimental research, and many NPs are still in the laboratory stage.

The application of NPs in cold ablation can be generally divided into two types: synergistic effect and protective effect, which are different in terms of the design requirements of NPs and the distribution in vivo. In the future, nano-cryosurgery may be assisted by a variety of NPs, viz, synergistic NPs are distributed inside the tumor while protective NPs are distributed around the tumor. In addition, many nano-positioning devices, such as puncture-designed 3D printed coplanar template (3DPCT) which currently used for tumor positioning before radioactive particle implantation may be used in cryosurgery. Prior to the cryosurgery, protective NPs can be punctured and injected around tumor to protect the surrounding healthy tissue by 3D printing coplanar template (3DPCT) and CT guidance. The NPs are able to assist the cryosurgery ice balls to cover the irregular edge of tumor. Then synergistic NPs will be introduced into the tumor tissue through the preset ablation site puncture or vascular intervention to perform cold ablation. This nano-cryosurgery technique can not only overcome the difficulties of cold ablation of irregular tumors but also increase the effect of cold ablation and reduce the damage to healthy tissue. This method may become the future research direction of nano-cryosurgery. Table 4 highlights some recent examples about NPs used in cryosurgery.

**Table 4:** Typical NPs platforms used in cryosurgery

NPs	Size (nm)	Thermal conductivity (W/m K)	Heat capacity (J/m^3^ K)	Benefits	Ref
PCM NPs	10–20	0.35	2.56 × 10^6^	Health tissue protection	[76]
Fe3O4 NPs	8–14	7.1	3.2 × 10^6^	More intracellular ice formation High thermal conductivity	[77]
Au NPs	3	297.7	2.2 × 10^6^	Good biological compatibility High thermal conductivity	[78]
HCPN-CG NPs	103.9 ± 1.5	None	None	Cold-responsive nanoparticle for controlled drug release NIR-induced photothermal effect	[79]
MgO NPs	50	34.3	3.2 × 10^6^	Nontoxic, biodegradable, and few side-effects	[74]

*HCPN-CG* H for hyaluronic acid, C for chitosan, P for PF127, N for PNIPAM-B, C for CPT, and G for ICG, *PCM* phase change materials

## Nanoparticles for PTT and PDT

**Fig. 6:**
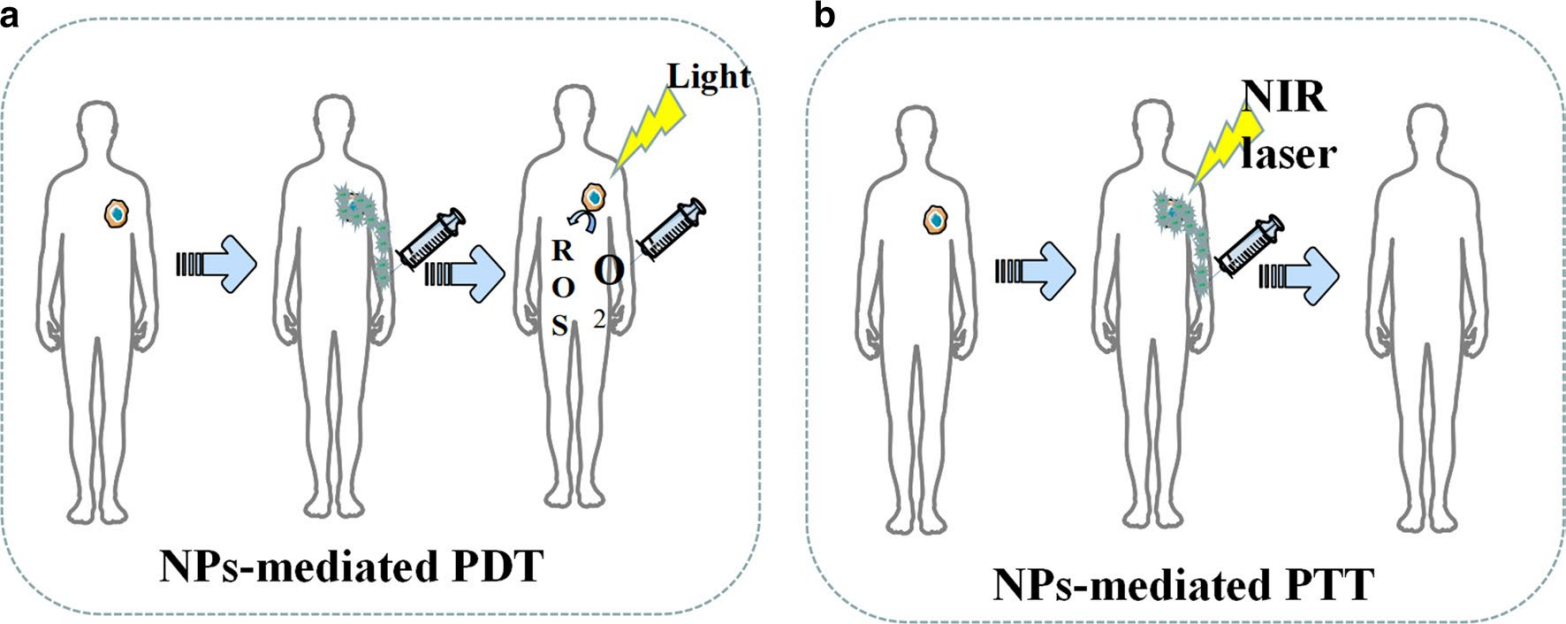
Diagrammatic illustration of NPs-mediated PDT and PTT. **a** NPs promote the generation of reactive oxygen. **b** NPs enhance tumor damage during PTT

At present, photothermal therapy (PTT) and photodynamic therapy (PDT) based on nanoparticles (NPs) have shown the virtues of strong efficacy, small invasion and mild adverse effects during tumor treatment (Fig. 6) [80]. In addition to killing tumor cells directly, fragments of dead tumor cells produced by PDT and PTT treatment can be used as potential antigens to trigger a continuous immune response, called photothermal and photodynamic immunotherapy [81]. Nanoparticles designed based on the PTT treatment concept are a new type of light-to-heat conversion nanomaterials, which can convert light energy into heat energy to kill cancer cells. Compared with traditional photothermal conversion materials, nanoparticles have many advantages. First, NPs can achieve the effect of tumor targeted aggregation through particle surface modification, which contributes to higher enrichment ability of target tumor [82, 83]. Second, nanoparticles have better imaging capabilities than traditional photothermal materials, which can be accurately positioned by CT, MRI and photoacoustic imaging [84, 85]. Targeted nanoparticles synthesized by Pan et al. can perform PTT under 0.2 W/cm^2^ NIR to induce tumor cell apoptosis by destroying the tumor cell nuclear DNA and inhibiting the DNA repair process [86]. Table 5 lists some recent examples about NPs used in PDT and PTT.

**Table 5:** Typical NPs platforms used in PDT and PTT

NPs Platform	Photosensitizers	*λ* (nm)	Size (nm)	Effects	Cell line	Ref
MnO_2_	Chlorin e6	660	3.94	Upregulating the secretion of IL-12,IFN-γ,TNF-αInducing decomposition of tumor endogenous H2O2 to relieve tumor hypoxia	4T1 murine breast tumor	[87]
Au-liposome	None	780	100 ± 6.5	The cytotoxicity was enhanced to 90% upon laser irradiation for a duration of 5 min	B16 F10 (melanoma)	[88]
Silica-coated TiN	None	785	80	High nitridation temperatures and long residence times lead to increased NIR light absorption	HeLa cells	[89]
Silica	Verteporfin	425	160–168	Inducing singlet oxygen release 30% reduction in cell growth	SK-MEL 28 (melanoma)	[90]
Graphdiyne	None	808	160	Higher cancer inhibition rate compared both in vitro and in vivo Biocompatibility and no obvious side effects	MDA-MB-231	[91]
RCDs	chlorin e6	671	3.7	Multimodal imaging capabilities Activating PTT and PDT at the same time	MCF-7,4T1 and Hela	[92]
CuSe	non-porphyrin containing COF	808	150	Activating PTT and PDT at the same time Enhancing therapeutic effect on killing cancer cells and inhibiting the tumor growth	HeLa	[93]
HSA	ICG and chlorin e6	808	120	Preventing the side effects of active Ce6 Activating PTT and PDT at the same time	PC3	[94]

*RCDs* amino-rich red emissive carbon dots, *COF* covalent organic framework, *ICG* indocyanine green, *HAS* serum albumin

In addition, some studies have found that nanoparticle-mediated PTT can reverse tumor multidrug resistance (MDR). The overexpression of drug transporters, multidrug resistance-associated protein 1 (MRP1), and p-glycoprotein (p-gp) are generally believed to cause MDR in various tumors [95]. For example, multifunctional light-triggered nanoparticles designed by Li et al. can inhibit the expression of MRP1 in PTT, which consequently reverses the drug resistance of A549R cells [96]. Wang et al. reported that both gold nanoparticles and carbon-based nanoparticles can overcome DOX resistance by promoting the expression of heat shock factor trimer in PTT, thereby inhibiting the generation of p-gp [97, 98]. Besides, nanoparticle-mediated PTT can also increase the effectiveness of chemotherapy by destroying the integrity of tumor cell membranes [99].

PDT is a treatment that uses the selective retention of photosensitizing substances (PSs) in tumor tissue under the activation of specific wavelength excitation light and the presence of molecular oxygen to produce singlet oxygen and other reactive oxygen species, which leads to tumor cell apoptosis and necrosis [100]. However, traditional PS has poor tumor targeting, poor solubility, and instability, which is vulnerable to the internal environment [100]. Nanoparticle carriers modified by targeted molecules can not only improve the stability and biocompatibility of PS but also deliver PS to target cells, which improves the efficacy and reduces adverse effects [100]. Additionally, some common nanomaterials, like gold nanorods, have excellent PTT effects themselves. For example, Vankayala et al. found that the exposure of gold nanorods to near infra-red light (915 nm) were able to efficiently induce the generation of singlet oxygen [100].

In recent years, the role of up-conversion (UC) nanoparticles in PDT has attracted much attention. The NPs can convert long-wavelength light excitation into multiple short wavelengths, which enables the UC to replace the traditional ps-dependent short-wavelength excitation light with the near-infrared light with strong tissue penetration ability [101]. For example, Li et al. developed dual-band luminescent lanthanide nanoparticles as a PS carrier. This UC nanoparticles rely on the excitation light wavelength of 808 nm to achieve image-guided PDT without affecting imaging signals [102].

Since most photosensitive materials utilized in the phototherapy are metals, the biocompatibility of NPs designed for inorganic nanomaterials like metal ions still needs to be improved.

NPs-mediated phototherapy is now credited for not only the effectiveness against tumor but also the potential for spare internal space of nanoparticles since the therapy only utilizes the physical properties of NPs skeleton. Therefore, NPs are often multifunctioned by PDT and PTT. In the future, such NPs may be designed as dedicated NPs for tumor stem cells that are not sensitive to chemotherapy. Tumor stem cells are dormant for a long time and have a variety of drug-resistant molecules, so it is difficult to kill them by conventional treatments like chemotherapy, whereas the light therapy is more effective by killing the tumor stem cells physically. In the future, nanophysical therapy may be used with many other techniques, such as the multifunctional NPs for photothermal therapy after cryosurgery. Multifunctional NPs mediated therapy can give full play to its characteristics of low side effects, strong local lethality, and tumor stem cell killing. In addition, because nano-physiotherapy has a local killing effect and can effectively kill tumor stem cells, it may become a treatment method for small metastases.

## Nanoparticles for Radiotherapy

Radiotherapy (RT) is a tumor treatment technique that kills local cells by ionizing radiation generated by rays and is currently an effective treatment for many primary and metastatic solid tumors [103]. Experiments prove that radiotherapy can effectively kill tumor stem cells [104].However, how to further improve the efficacy of radiotherapy is still a serious challenge. In recent years, nanoparticles in the field of radiotherapy have demonstrated strong radiosensitization capabilities, tumor-targeted delivery capabilities of radiosensitizing drugs, and imaging guidance enhancement capabilities [105]. At present, the most popular nanoparticles are made by high Z (atomic number) metal materials, which are featured by chemical inertness and strong radiation absorption capacity. They produce various reactions such as photoelectric effect and Compton effect after absorbing radiation, thereby releasing a variety of particles such as optoelectronics, Compton electrons, and Auger electrons. These electrons react with organic molecules or water in tumor cells to generate a large number of free radicals, leading to synergistic chemotherapy [106]. Common chemotherapy-sensitized NPs are currentlycategorized as precious metals, iron oxides, and semiconductors in terms of materials.

Precious metals NPs are made of high atomic number metal materials such as gold, silver, gadolinium, hafnium, platinum, bismuth, etc. [107]. Among them, gold nanoparticles have become the most popular NPs due to their good biocompatibility, chemical stability, and relatively strong photoelectric absorption coefficient [108]. In 2000, Herold et al. discovered the chemosensitizing ability of gold nanoparticles in kilovoltage X-rays. Nowadays, the specific mechanism of chemosensitization of gold nanoparticles is not yet clear, and the mainstream view believes that it depends on the photoelectric absorption capacity of high atomic number [109]. In addition to this, there are studies suggesting that the presence of gold nanoparticles improve the chemical sensitization of DNA to radiation, which increases the DNA damage induced by ionizing radiation (IR). At the same time, gold NPs can catalyze the mechanism of radiotherapy sensitization such as free radical production [105]. For instance, Liu found that AuNPs could significantly increase the production of hydroxyl radicals as well as the killing effect of x-rays and fast carbon ions on cells [110]. The hypothesis of the chemotherapy sensitization mechanism of other precious metals is similar to that of gold nanoparticles. Particularly, platinum NPs have an anti-tumor effect due to the inherent nature. Consequently, platinum NPs are expected to play the role of chemotherapy and radiotherapy simultaneously. However, the number of relevant research reports is insufficient, and the sensitizing effect of platinum NPs is also questionable. For example, Charest et al. reported that liposomal formulation of cisplatin was able to increase the uptake of platinum by tumor cells, and could enhance the killing of F98 glioma cells by γ-rays at the same time [111]. On the contrary, Jawaid et al. reported that platinum NPs would reduce the generation of reactive oxygen species (ROS) and the efficacy of radiotherapy during chemotherapy [112].

Iron oxide nanoparticles (IONs), especially the superparamagnetic magnet Fe_3_O_4_, have shown great potential in image-guided tumor radiotherapy because they are capable of enhancing the dose of radiotherapy and MRI imaging, whereas its sensitization mechanism is not clear yet. Its sensitization mechanism is not yet clear. Some studies believe that iron oxide NPs mainly catalyze the generation of ROS through Fenton's reaction and Haber–Weiss reaction. Then the highly reactive ROS will kill tumors [112–115]. Other studies propose that the mechanism depends on the radiation sensitization and synergistic effects of magnetic nanoparticles. As Khoei reported, iron oxide NPs can improve the radiosensitization of prostate cancer cells in vitro [116]. Huang et al. pointed out that cross-linked dextran-coated IONs (CLIONs) could be internalized by HeLa cells and EMT-6 mouse breast cancer cells, which enhances radiation therapy [117]. Although the synergistic effect of iron oxide NPs is obvious, its biological safety still needs to be improved. Many studies have proved that the biocompatibility and chemical stability of iron oxide NPs are questionable, and it has certain toxicity [118].

Semiconductor NPs like silica NPs have also been found to have a synergistic effect on radiotherapy. For instance, Zhang et al. used flow cytometry analysis and MTT experiments to find that mesoporous silica NPs can effectively enhance the radiotherapy of glioblastoma [119]. He et al. reported the mechanism of radioactive enhancement of silica NPs. He found that under X-ray irradiation, silica nanoparticles could produce fine hydroxyl radicals, which can effectively kill tumor cells [120].

At present, although many experiments have confirmed that NPs were able to sensitize radiotherapy, the specific mechanism of sensitization is still unclear, which hinders the development of new sensitized NPs. There are some doctrines like sensitizing chemotherapy that promotes free radical production. Nevertheless, there is a lack of a quantitative relationship among the amount of free radical production, radiation intensity, and physical data of nanoparticles. In addition, most sensitized NPs are made of high atomic number metals. These metals have many disadvantages in human body such as difficulty in self-metabolism and biodegrading. Meanwhile, long-term accumulation of the metals will produce toxicity, which limits the safe use of radiosensitized NPs. Moreover, compared with the radiotherapy sensitization NPs, fewer studies focused on NPs which can prevent the adverse reactions of radiotherapy and protect healthy tissues. The research on radiotherapy protective NPs is short in quantities.

In the future, searching for NPs material that can be metabolized by the kidney, biometabolized, biocompatible, stable in physicochemical properties, and inherently less toxic, or looking for surface modification that can help the body metabolize NPs may become a research direction for sensitized NPs. Moreover, although there have been many NPs studies on multi-function, namely simultaneous sensitization of radiotherapy and chemotherapy, there are still many potentials in this field, which are worthy of focus in the future. The development of protective NPs that can protect normal tissues around radiotherapy and alleviate poor defense against radiotherapy may also become a research direction.

## Conclusion

The poor curative effect, inefficient targeting ability, various side effects, and potential biological risk are some of the unfavorable attributes of conventional cancer therapy and diagnosis. In recent years, advanced nanotechnology and molecular cell biology have promoted the applications of NPs in cancer field. Not only metal NPs, but also many lipid, nucleic acid and silicon NPs showed evident outperformance in cancer diagnosis and treatment.. Moreover, new generation of NPs is no longer limited to solo but multiple functions. For example, gold-coated poly(lactic-co-glycolic acid) (PLGA) NPs equipped with PD-1 blockers which were designed by Luo et al. can not only target drug delivery but also mediate PTT therapy [121]. (Pd @ Au) / Fe3O4 Spirulina NPs with doxorubicin created by Wang et al. demonstrated the functions of photothermal therapy, delivery of chemotherapy drugs, and magnetic field control in cell experiments [122]. Multifunctional nanoparticles will become the trend of future research.

At present, we find that most of the nanoparticles only stay in vivo and in vitro stage. According to this review, we think the following reasons hinder the clinical application of NPs.

(i) Lack of injection routes and methods

Most NPs are injected into body via puncture or intravenous injection. Therefore, the blood flow will take away NPs, making NPs difficult to stay in the target area for a long time, which leads to just few NPs that can be uptaked by tumor cells. Low-concentration drugs cannot produce the expected therapeutic effect, and low-concentration NPs also affect the physical killing effects of PDT, PTT, cryosurgery, and radiotherapy. In our opinion, magnetic NPs platform may be a solution. There have been many in vitro and in vivo experiments that have proved the feasibility of using the three-dimensional magnetic field to control the movement of NPs against blood flow [122–125]. However, how to solve the interference of the human body to the magnetic field, how to solve the impact of blood cells colliding with NPs, and how to control a large number of NPs in a group are still in discovery.

(ii) Difficulty in localization of NPs in vivo

Compared with the human body, the size of NPs is too tiny. Even if NPs are loaded with fluorescent proteins, it is still difficult for conventional imaging equipment (CT, X-ray, MRI) to locate the NPs in the human body in real time. To deal with this challenge, photoacoustic computed tomography (PACT) may be a solution. Photoacoustic computed tomography (PACT) has attained high spatiotemporal resolution (125-μm in-plane resolution and 50-μs frame^−1^ data acquisition), deep penetration (48-mm tissue penetration in vivo), and anatomical and molecular contrasts [126]. Because of excellent performance, PACT has great potential in NPs localization imaging in vivo. The PACT-guided microrobotic system designed by Wu et al. has achieved controlled propulsion and prolonged cargo retention in vivo of NPs with a diameter of 50 μm [127]. Although the current resolution and deep penetration of PACT are still insufficient, it is superior to conventional imaging equipment (CT, X-ray, MRI) in terms of NPs imaging positioning.

(iii) Difficulty of degrading in the human body

Although NPs are made of high biosafety materials, there is still a risk of damages to liver, kidney, and other organs if they stay in the body for a long time and cannot be degraded or excreted The use of materials that will be disintegrated after near-infrared light irradiation to fabricate NPs may be a solution to this problem. Recently, more and more NPs have been produced by these materials. Such NPs mediate PTT while loading drugs, meanwhile, the substances produced by the disintegration of NPs can be rapidly metabolized by the human body. In addition, the use of more biocompatible and degradable materials for nanoparticle preparation is also a solution. For example, the surface of chitosan is positively charged and can be broken down by the colonic flora, which facilitates interaction with specific tissues and can be metabolized by the body. The biocompatibility and degradability of chitosan has been proven to be non-toxic at appropriate drug concentrations [128].

(iv) Difficulty in avoiding mononuclear phagocytic system (MPS)

In biofluids, NPs will adsorb proteins to form a corona layer referred to as “protein corona” in a broader sense giving biological identity to NPs and alters their biological characters, which will attract MPS especially macrophages to uptake NPs [129]. In order to avoid being uptaken by MPS, various polymer coatings such as forpolyether, polybetaine (PB) and polyolhave were investigated to cover NPs. For example, polyglycerol-grafting NPs are able to evade macrophage uptake by reducing protein adsorption [130]. In addition, there are two types of tumor-associated macrophages (TAM), M1 and M2. M1 macrophages inhibit tumor growth while M2 macrophages promote tumor growth. Therefore, no longer avoiding macrophages, but designing NPs targeted by macrophages, by regulating the function of macrophages, and even using macrophages as new drug carriers to exert anti-tumor effects may become a novel solution. At present, common design strategies for such NPs include inhibiting macrophage recruitment, depleting TAM, reprogramming TAMs, and blocking CD47-SIRPα pathway [131]. Among them, following the design concept of reprogramming or blocking CD47-SIRPα pathway, NPs that repolarize M2 macrophages to M1 type have made a breakthrough in vivo experiments [132].

Considering the above difficulties and referencing to advanced researches, we come up with a new possible design of NPs. The NPs skeleton is made of pyrolytic material (spirulina, exosomes, et al.). Then, photothermal materials (Au, Pd, etc.) are deposited on the NPs skeleton through electroless plating. After that the superparamagnetic iron oxide will be loaded on the surface of NPs through the sol–gel method. Then, suitable polymers (polybetaine, polyglycerol, etc.) will coat the NPs. Finally, drug (like doxorubicin) will be loaded on the NPs. Afterwards, under the guidance of PACT, NPs will be injected into the upstream of tumor supplying blood vessel, and the tumor will be irradiated with NIR. At the same time, three-dimensional magnetic field control is given to maximize the accumulation of NPs at the tumor site. Through this design, a large number of NPs will accumulate at the tumor site to ensure the drug concentration and PTT effect. At the same time, most NPs will be decomposed at the tumor site, and only a small number of NPs will circulate in the body.

Nowadays, anti-tumor therapy with NPs as the main body is still in the exploratory stage, and related technologies and equipments need to be invented, so it is unlikely to be clinically used in the short term. However, NPs can change part of the function or structure of many actual technologies. The upgrade of actual technologies is expected to be applied in clinic quickly, which contributes to upgrading the diagnosis and treatment of tumors in consequence. For example, NPs can help to develop electrochemical devices based on the interaction between ions and conductive polymers, such as organic electrochemical transistors (OFETs), electrolyte gated field-effect transistors (FETs), fin field-effect transistor (FinFETs), tunneling field-effect transistors (TFETs), electrochemical lab-on-chips (LOCs) [133]. These electrochemical devices are widely used in various tumor testing and diagnostic equipment. The use of NPs can help improve the accuracy of the equipment and reduce the detecting time. Many studies indicate that medical equipment using electronic components upgraded by NPs have been applied clinically [133–136].

Based on the evidence cited above, future research of NPs may not only focus on NPs themselves but also consider a feasible administration and efficacy assessing platform. In addition, the platform needs to be able to monitor immunotoxicity, the long-term toxicity, and neurotoxicity of NPs. As nanotechnology develops, if these problems were solved, NPs would be an ideal approach to upgrade cancer therapy and diagnosis.

## Data Availability

All data generated or analysed during this study are included in this published article.

## Abbreviations

NPs: Nanoparticles
PDT: Photodynamics therapy
PTT: Photothermal therapy
SPR: Plasmon resonance effect
Au NRs: Gold nanorods
SI-ATRP: Surface-initiated atom transfer radical polymerization
NIPAAM: N-isopropylacrylamide
US: Ultrasound
MSNs: Mesoporous silica nanoparticles
USMO: Ultrasmall manganese oxide
GEM: Gemcitabine
OINPs: Oxygen/indocyanine green-loaded lipid nanoparticles
PA: Photoacoustic
MPI: Magnetic particle imaging
MRI: Magnetic resonance imaging
SPIO: Superparamagnetic iron oxide
USPIO: Ultra-small SPIO
OCT: Optical coherence tomography
MMOCT: Magnetomotive optical coherence tomography
mAb: Monoclonal antibody
DOX: Doxorubicin
5-FU: 5-Fluorouracil
FA: Folic acid
PTX: Paclitaxel
ROS: Reactive oxygen species
EPR: Enhanced permeability and retention effect
EGFR: Epidermal growth factor receptor
BHC: Berberine hydrochloride
AFP-1: Antifreeze protein
PCMs: Phase change materials
3DPCT: 3D printed coplanar template
RCDs: Amino-rich red emissive carbon dots
COF: Covalent organic framework
ICG: Indocyanine green
HSA: Serum albumin
MDR: Multidrug resistance
MRP1: Multidrug resistance-associated protein 1
p-gp: P-glycoprotein
PSs: Photosensitizing substances
UC: Up-conversion
RT: Radiotherapy
PLGA: Poly(lactic-co-glycolic acid)
PACT: Photoacoustic computed tomography
MPS: Mononuclear phagocytic system
PB: Polybetaine
TAM: Tumor-associated macrophages
OFETs: Organic electrochemical transistors
FETs: Electrolyte gated field-effect transistors
FinFETs: Fin field-effect transistor
TFETs: Tunnelling field-effect transistors
LOCs: Electrochemical lab-on-chips
